# Inhibitors and Supporters of Policy Change in the Regulation of Unhealthy Food Marketing in Australia

**DOI:** 10.34172/ijhpm.2024.7405

**Published:** 2024-03-13

**Authors:** Yandisa Ngqangashe, Sirinya Phulkerd, Ashley Schram, Jeff Collin, Carmen Huckel Schneider, Anne Marie Thow, Sharon Friel

**Affiliations:** ^1^School of Regulation and Global Governance (RegNet), College of Asia and the Pacific, Australian National University, Canberra, ACT, Australia.; ^2^Institute for Population and Social Research, Mahidol University, Salaya, Thailand.; ^3^School of Social and Political Science, University of Edinburgh, Edinburgh, UK.; ^4^Menzies Centre for Health Policy and Economics, Sydney School of Public Health, University of Sydney, Sydney, NSW, Australia.

**Keywords:** Food Marketing Policy, Australia, Food Advertsing Regulation

## Abstract

**Background:** Evidence on the impact of policies that regulate unhealthy food marketing demonstrates a need for a shift from pure industry self-regulation toward statutory regulation. Institutional rules, decision-making procedures, actor practices, and institutional norms influence the regulatory choices made by policy-makers. This study examined institutional processes that sustain, support, or inhibit change in the food marketing regulation in Australia using the three pillars of institutions framework – regulatory, normative, and cultural cognitive pillars.

**Methods:** This was a qualitative study. Twenty-four in-depth semi-structured interviews were conducted with industry, government, civil society, and academic actors who are involved in nutrition policy in Australia.

**Results:** The regulatory pillar was perceived to inhibit policy change through the co-regulation and self-regulation frameworks that assign rulemaking, monitoring and enforcement to industry bodies with minimal oversight by regulatory agencies and no involvement of health actors. The normative pillar was perceived to provide pathways for comprehensive statutory regulation through institutional goals and norms for collaboration that centre on a whole-of-government approach. The framing of food marketing policies to highlight the vulnerability of children is a cultural cognitive element that was perceived to be essential for getting support for policy change; however, there was a lack of shared understanding of food marketing as a policy issue. In addition, government ideologies that are perceived to be reluctant to regulate commercial actors and values that prioritize economic interest over public health make it difficult for health advocates to argue for statutory regulation of food marketing.

**Conclusion:** Elements of all three pillars (regulatory, normative, and cultural-cognitive) were identified as either inhibitors or pathways that support policy change. This study contributes to the understanding of factors that inhibit policy change and potential pathways for implementing comprehensive statutory regulation of unhealthy food marketing.

## Background

**Key Messages**

**Implications for policy makers**

- The findings of this study highlight a need for increased participation of health actors in the regulation of unhealthy food marketing at the Federal Government level. This can be achieved through intersectoral collaboration and whole-of-government approaches that have been successful in achieving comprehensive regulation of unhealthy food marketing at the State/Territory government levels.
- Government institutional processes should be protected from affording commercial interests more influence than public health actors.
- A shared understanding of unhealthy food marketing among health advocates is crucial to advancing comprehensive food marketing regulation efforts.

**Implications for the public**

Policies that regulate unhealthy food marketing to children have potential to reduce the burden of obesity and improve health outcomes. Currently, there are institutional factors that inhibit the implementation of mandatory policies that regulate the marketing of unhealthy food marketing to children. This study has identified these factors to inform the development of targeted strategies for advancing the development of comprehensive unhealthy food marketing policies. This study has also identified institutional factors that have led to the successful development of food marketing policies at State/Territory government levels. These enabling factors which include intersectoral collaboration and whole of government approaches can be adopted by Federal government and other State/Territory government institutions to advance the regulation of unhealthy food marketing.

Marketing is central to food industry strategies to shape the acceptability and desirability of ultra-processed foods, thereby normalizing consumption and increasing demand for such products. Consequently, people’s diets have shifted towards highly palatable, mass-produced, heavily marketed ultra-processed food products.^1-3^ These dietary patterns contribute to increased weight gain and the global burden of non-communicable diseases.^4,5^

 There have been numerous calls to implement policies that restrict the marketing of unhealthy food to improve the supply, availability, and consumption of healthy food, to counter the rising burden of non-communicable disease.^6-8^ The World Health Organization (WHO) set of recommendations on marketing foods and non-alcoholic beverages to children recommends that governments regulate a broad range of commercial communication to children, including advertising on broadcast media, promotions, and places where children gather.^6^ Despite such calls, the progress on government regulation of marketing is slow and existing policies are not comprehensive enough to protect children from all forms of food marketing communication.^9-11^

 Globally, policies that regulate the marketing of unhealthy food marketing to children are dominated by industry self-regulation approaches, whereby the food and advertising companies design, monitor, and enforce their own rules with no formal oversight from the government or other public health interest groups.^9,11^ Statutory regulation by governments can occur using a range of laws and regulations, such as food and nutrition laws, consumer protection laws and broadcasting laws, as observed in the United Kingdom, Ireland, and Quebec (Canada).^12^ In some jurisdictions that have implemented statutory policies, available evidence shows that multi-pronged government-led regulation is more effective in reducing exposure to unhealthy food marketing than industry self-regulation.^11,13-17^ Despite the better performance of statutory regulation models and the well-documented failures of industry self-regulation approaches to reduce exposure to unhealthy food marketing,^18-21^ the shift from self-regulation to government-led regulation is still convoluted.

 Studies on the current and past policies to restrict unhealthy food marketing to children in Australia have found these policies ineffective in reducing exposure and the prevalence of children to unhealthy food marketing, as children were still highly exposed to unhealthy food marketing.^22-24^ Challenges identified in previous studies include a lack of oversight by regulatory institutions and measures to ensure accountability in industry initiatives.^12^ Chung et al highlighted the challenges with regulating the marketing of unhealthy foods as the perception of roles between state and federal government institutions, the food industry’s influences and a lack of evidence on the effectiveness of regulations.^25^ Increasingly, public health scholars have been examining the drivers of policy and regulatory change. Studies of nutrition policy identified the role of actor interests, political and institutional contexts, framing issues, and availability of resources as the essential factors in understanding policy change.^26-28^ Political, legal, economic, cultural, and ethical contextual factors influence not only the choice of regulatory instruments but also the delegation of responsibility for regulating food marketing across different institutions.^29^ Specific to the delegation of responsibilities between institutions, this is a crucial investigation to understand food marketing regulation in the federated government contexts such as in the United States, Canada, India, and Australia. These countries shared the responsibility of regulating food marketing between federal and state governments and by different institutions with varying mandates and interests. Box 1 outlines an in-depth summary of the delegation of food marketing policies in Australia.

**Box 1. A Summary of the Food Marketing Regulatory Context in Australia**

The governance of food marketing is split between the Federal Government and comprises eight states/territories, regulatory agencies and industry bodies. Broadcast media is regulated at the Federal government level in a co-regulation arrangement between the ACMA and industry bodies AANA and the Outdoor Media Association. ACMA’s role is mainly in relation to the CTS 2009, which applies to commercial television broadcasters.^30^ In November 2020, the CTS were revised 2020 and provided guidelines for marketing during C and P-rated children’s television programming on free-to-air TV. The CTS are mandatory and apply to programs that are classified as C (children) and P (pre-school children). The rules are generally applied to all advertising, including foods.^31^

The rest of the rules on food marketing are principally in self-regulatory industry codes of practice developed and administered by the AANA, a peak body for advertisers. Before November 2021, there were three voluntary codes from the food and advertising industries. The food industry codes included the Australian Food and Grocery Council’s Responsible Marketing to Children Initiative and the Quick Service Restaurants Initiative. In 2021, the Responsible Marketing to Children Initiative and the Quick Service Restaurants Initiative were discontinued as the AANA took over the administration of all food and industry codes. The AANA instituted the Food and Beverages Advertising and Marketing Communications Code and the AANA Code for Marketing and Advertising Communications to Children, which currently apply to marketing across all media, including online. There is also a voluntary code by the Outdoor Media Association (Health and Wellbeing Policy) that restricts the marketing of “discretionary” foods within a 150-metre sightline of a primary or secondary school.^32^

At the state/territory government level, the regulation of food marketing in public spaces such as billboards, schools, sports centres, and hospitals is the responsibility of state/territory governments and is under the jurisdiction of the departments or institutions that own those spaces. The progress in the institution of unhealthy food marketing varies across different states. For instance, in the Australian Capital Territory, the marketing of unhealthy food is banned in public spaces, including billboards and public transport, while in New South Wales and Western Australia, the advertisement of unhealthy food marketing is banned on properties that the Department of Health owns.

-----------------

Abbreviations: ACMA, Australian Media and Communications Authority; AANA, Australian Association of National Advertisers; CTS, Children’s Television Standards.

This study examined the institutional processes that shape food marketing policies in Australia, building on previous studies that primarily focused on broadcast media.^12,22-24^ We aimed to understand how institutional processes, including rules, decision-making procedures, actor practices, and norms, create and maintain the current regulatory approaches and to explore how policy change towards a comprehensive statutory regulation of food marketing could be facilitated.

## Methods

###  Theoretical Framework 

 In this study, institutional theory framework is used to examine how decision-making procedures, norms, and actor practices restrict or enable policy change in food marketing regulation in Australia. Institutions are defined as formal and informal rules that guide how policy actors behave, including the policy changes they make.^33,34^ Institutions can restrict behaviour by prescribing what legally, socially and morally appropriate action is and empower actors by providing guidelines and resources for acting.^34^ This study drew specifically from Scott, who posits three pillars (regulatory, normative, and cultural-cognitive) that make up the fundamentals of institutions. First, the regulatory pillar entails processes that include the capacity to create, monitor and enforce rules. While rules can be informal, regulatory systems are characterized by highly legalized rules with obligation, precision, and delegation of third parties to monitor and enforce. Under the regulatory pillar, systems change or remain the same out of legal obligation. Second, the normative pillar emphasizes values and norms that define goals and prescribe appropriate action to achieve these goals.^34^ From a normative perspective, policy change is driven by moral obligation and duties or responsibilities.^34^ Third, the cultural-cognitive pillar emphasizes values, beliefs, and assumptions about an issue. Under this pillar, change is driven by actors’ desires, reflecting their internalized values on the issue.^34^ In this analysis, these three pillars are used to help the understanding of institutional factors that drive and/or sustain change in the regulation of food marketing policy in Australia.

###  Recruitment Process 

 The interviews were undertaken with actors from government departments of health, statutory health promotion bodies, technical experts, civil society, and industry (media, advertising, and food industries) between the end of May 2020 and March 2021. The government interviewees included stakeholders from the Australian Federal Government Department of Health and state/territory health departments. Purposive sampling, targeting interviewees involved in and who have knowledge of the subject matters was used^35^ (ie, food marketing and/or nutrition policy in Australia). First, stakeholder mapping exercise that identified the different organizations involved in food marketing policies was conducted. Names and contact details of potential interviewees were obtained from the websites of the identified organizations. Technical experts were identified through publications on food marketing and food marketing regulations in Australia. Additional interviewees were identified through snowballing.

###  Data Collection or Interview Protocol 

 Participation was voluntary, with interviewees who agreed to participate would respond via emails that included a signed consent form. This was a qualitative study. Semi-structured were conducted using an interview guide that was developed iteratively by a team of seven researchers with interdisciplinary expertise in public health, health policy, food regulation, and political sciences. The interview guide was developed before the interviews based on three core lines of enquiry (*i*) Who is involved in the governance system related to food marketing policies in Australia? (*ii*) How are they governing? and (*iii*) What could good governance for nutrition look like? The institutional analysis of the current study was on who is governing and how they are governing. The interviews explored actors’ organizational roles, priorities and objectives related to food marketing regulation and the formal and informal processes actors use to achieve their objectives. This was done to examine how existing institutional factors impact the ability of actors to make effectual decisions and how the agency is exercised within and constituted by these institutions.

 All interviews were conducted using Zoom. To protect the participants’ anonymity, the interviewers only recorded audio content, and at the end of the interview, the participants’ audio file was encrypted using a unique code before transcription. The anonymized audio files were transcribed verbatim, and the transcripts were quality-checked by the researchers (YN, AS, and SP). The cleaned transcripts would be sent to the corresponding participants for verification only when requested. The researchers continued recruiting participants until reaching data saturation, which was indicated by regularly getting the same responses from the participants.

###  Data Analysis

 The data were analyzed thematically with QSR International NVivo version 13 using a combination of deductive and inductive analysis.^36^ The coding process was iterative and involved the whole research team. First, five transcripts were read by the research team, which consisted of four chief investigators and three early career researchers with expertise in food policy, to identify the initial codes. After coding, the emerging themes were organized into Scott’s three institutional pillars – regulatory, normative, and culturally cognitive. One researcher then coded the rest of the interviews (YN), adding codes as they emerged and getting feedback from the team. Additionally, five transcripts were randomly selected to be re-coded by one of the chief investigators (CHS).

## Results

###  Participant Characteristics 

 Thirty-eight participants were identified, 26 agreed to participate, and 24 were interviewed (Table). There were two stakeholders from the Federal Department of Health and one written response from the Department of Communications. There were six state government interviewees. Of these, five were from the health department, and one was from the Department of Sports department. There were two interviews from statutory health promotion agencies that operate at the state level and three civil society interviews representing media and health. There were also four food and media industry actors, five technical experts and one politician (former senator). There was one stakeholder from one federal government actor (communication) who provided a written response that explained the institution’s limited role in the regulation of food marketing, while one participant agreed to be interviewed but did not participate.

**Table:** List of Stakeholders Who Were Interviewed

**Actor Group and Subgroup**	**Participants**
Government	
Federal Health	2
State Health	5
State Sports	1
State statutory bodies (health promotion agencies)	2
Media*	1
Industry	
Food and beverages Industry	2
Media and Advertising Industry	2
Civil society	
Health	2
Media	1
Technical expert	5
Politician	1
**Total**	**24**

* A written response actor.

 In the following result section, the next section presents how factors in the regulatory, normative, and cultural-cognitive institutional pillars drive, sustain and or inhibit changes in food marketing policies in Australia as described by the interviewees. The section also describes how these three pillars intersect and reinforce one another in the food marketing policy process.

###  Regulatory Pillar

 The regulatory pillar entails laws, policies, and rules and how they are instituted, monitored, and enforced. This pillar primarily influences food marketing policy change through the regulatory frameworks that guide who has the power to set and enforce rules and how regulation of food marketing is delegated between different organizations and through state and federal government inter-governmental structures.

####  Regulatory frameworks 

 The federal government Department of Health interviewees described their limited involvement in the regulation of food marketing. Notably, there is no mandate from the Food Minister’s Meeting (a platform to develop food regulations for Australia and New Zealand) to restrict unhealthy food marketing to children. Although Food Standards Australia New Zealand (FSANZ) governs food advertising, the focus is mainly on tackling misleading food advertising while protecting children from the harmful impacts of food marketing, which was often treated as a separate policy issue.

 “*Food marketing is regulated by the communications portfolio. So, we do not regulate food marketing. The food regulation system does not actually regulate advertising. That’s not something that we work on as a big focus” *(Federal government official).

 While the Department of Health may have opportunities to make submissions to the Australian Communications and Media Authority (ACMA) during the development of legislation for marketing to children, there is no collaboration or common mandate related to the marketing of unhealthy food to children. The ACMA Children Services Standards do not specifically refer to food. From the perspective of the regulatory pillar, the co-regulatory approach between the ACMA and the Broadcasting industry, the self-regulation approach by the Australian National Association of Advertisers (AANA), as well as the absence of food marketing from the mandate in the food regulation system constrains the involvement of the Department of Health actors.

 Self-regulation, which entails the food and advertising industry designing, administering, monitoring, and enforcing their own unhealthy food marketing codes, is highly contested by state government Departments of Health, technical experts, and civil society actors. These contestations led to the lack of stakeholder participation during the policy consultation stage and the generation of research evidence to highlight the ineffectiveness of self-regulation.

 “*We’re not putting anything into [the policy consultation] because we don’t think the industry should be setting their own rules [referring to an industry self-regulation review]” *(Civil society actor).

 The main issues of contention were the lack of oversight from government entities like ACMA and the perceived conflict of interests associated with the industry monitoring and enforcing its own rules. Technical experts, civil society and some Department of Health officials argued that self-regulation ineffectively reduced children’s exposure to unhealthy food marketing but was portrayed as a ‘feeling good’ perception. Extending from this perspective, self-regulation was instrumental in maintaining the status quo.

 “*The whole phenomenon of industry self-regulatory codes…the fox guarding the henhouse…that’s seen globally, and it’s an easy [way] out for governments because they say, “Well, the industry is taking care of that. That’s fine” *(Technical expert).

####  Federal and State/Territory Intergovernmental Process

 Government interviewees described formal processes that different state/territory governments used to influence policy at the national level. This was primarily observed through the Council of Australian Governments (COAG) (disbanded in May 2020 around the beginning of the interviews). The Council served as an intergovernmental organization with representatives from all state/territory and federal level governments. State/Territory governments were able to put issues onto the COAG agenda, and policy decisions depended on consensus being reached by the different governments. Participants revealed that the COAG Health Council had an interest in obesity, which led to a national interim guide to reducing the marketing of unhealthy food to children. The interim guide was developed to help state governments with the regulation of marketing in school environments and sporting facilities. Pre-existing policy priorities for regulating unhealthy food marketing in some states and existing programs on school nutrition policies contributed to the advancement of food marketing policies.

 “*The ACT government had already announced that they were going to remove junk food advertising from their fleet of buses; they had an imperative to do that type of work, and New South Wales were doing it around their Premier’s objective” *(State government official).

 Despite the COAG initiatives, state/territory level actors reported feeling constrained by a lack of action at the Federal government level, as most advertising was from the national level. Various interviewees from state/territory governments’ health departments reported that the process of developing the National Preventative Health and the National Obesity Strategies might offer potential ways of improving their influence at the national level for policy reforms to regulate unhealthy food marketing to children.

 “*So, the National Obesity Strategy has a working group that includes representatives from all the Departments of Health, from all the states and territories…it’s a start [to push food marketing agenda]” *(State government official).

###  Normative Pillar 

 Scott’s institutional normative pillar relates to norms that guide the behaviour of different actors. In the interview data, this was articulated in terms of institutional goals and, priorities and norms for collaboration and consultation.

####  Goals and Priorities 

 In 2017, the Australia and New Zealand Ministerial Forum on Food Regulation published three priorities, including reducing chronic diseases related to overweight and obesity. Federal government officials viewed the priorities as a good opportunity for bringing food marketing into the policy agenda and shaping the activities of the Forum between 2017 and 2021.

 “*The Forum agreed to a suite of activities…looking at reducing overweight and obesity-related chronic conditions…the Health Star Rating (Australian front-of-pack labelling system) is a good example of what’s come through there (referring to the Food Regulation Ministerial Forum)…there is a possibility for some of the food marketing policy to come through there” *(Federal government official).

 In the four state/territory government interviews, food marketing policies had been highlighted as a priority within the state/territory Premiers’ office [head of government] and also as a part of the state Department of Health’s strategy. Through these two state/territory level institutional pathways, the regulation of food marketing became embedded in the policy package to achieve state/territory health goals.

 “*Our priorities are focused firstly on healthy weight because that’s the [state] government priority of the day. However, … policy implementation is always subject to the priorities of the government of the day” *(State government officials).

 Policy implementation was also enabled by the intersectoral collaboration between the departments, especially involving those with jurisdiction over their own spaces.

 “*These kinds of policies: removing junk food ads from public transport requires a decision, not just by the health minister, but it also requires a decision from the transport minister” *(State government official).

####  Norms for Collaboration and Consultation

 Food marketing policy change is shaped by norms that guide how the institutions that regulate food marketing collaborate and consult, particularly at the state/territory government level. States and Territories such as Queensland, Western Australia and the Australian Capital Territory had broad goals that set norms for intergovernmental collaboration. For example, the Australian Capital Territory had a “healthy weight initiative” led by an intergovernmental structure with chief ministers, which aimed to address increases in overweight and obesity and reduce the risk of cardiovascular disease. While the Department of Health contributed to the design of food marketing regulations, the policy ownership was shared with other departments, such as education and transport. Food marketing, therefore, became less of a “health issue,” and this was seen as an enabler by a few non-health government officials for advancing food marketing policy.

 “*It’s the whole of government policy; the Premier and Cabinet are the owners of that policy, and we are the technical experts that support that. And that’s a far, far stronger position to be in than having the Department of Health [to] own something”* (State government official).

 Another normative element that emerged was related to how industry actors accessed policy-makers and how the government responded to public health advocates and industry actors when the issue of legislating food marketing was raised. It was perceived as ‘normal’ for the industry to have easier and more immediate access to policy-makers compared to public health experts.

 “*A lot of their key stakeholders [referring to food and advertising industry actors] is that they can just demand meetings with ministers like that. They don’t have to wait in line like public health people, and they can just front up and say, ‘Right, I need to talk to you’” *(Technical expert).

 Civil society and technical experts argued that it had become a norm for the government to automatically accept industry efforts as adequate regardless of the views of public health advocates.

 “*The (government in power) does believe the industry when they are doing something about food marketing – So I think politicians are quite happy with the first information they get from the industry to say that it’s all right, we’re doing a good job” *(Civil society actor).

 Technical experts and civil society groups that have an interest in the legislation of food marketing had strong stances and argued on the appropriateness of industry involvement and related collaborations. For example, when the AANA released a review on food marketing codes in 2021, one of the civil society organizations led a boycott with support from other civil society groups and public health academics. In addition, one civil society interviewee alleged the misleading claim of the consultation processes by industry actors.

 “*We also had a bad experience with the Outdoor Media Association, who actually put out a release that said that they had consulted with (refers to the organization). They used the name to say that we had been consulted when they put out their policy, when in fact we hadn’t” *(Civil society actor).

 One state/territory department of health actor mentioned that they normally do not collaborate with industry except during policy implementation.

 “*…our policy is that we would not involve industry unless the discussions were about the implementation of the policy. We would not involve industry in the development of a policy”* (Government official).

###  Cultural-Cognitive Pillar 

 Scott’s institutional cultural-cognitive pillar emphasizes actors’ subjective interpretations of policy issues, beliefs, and external cultural frames that they use to shape policy discussions. In the regulation of food marketing, the elements of the cultural-cognitive pillar that emerged from the interviews included values that prioritize economic interests over health, actors’ ideas, and beliefs about regulating corporate activity, perceptions of food marketing as a problem, and the framing of food and policy solutions.

####  Values That Prioritize Economic Interests Over Public Health 

 A majority of actors from civil society, technical experts and state/territory government indicated that the mere mention of jobs and the economic implications by industry actors was enough to result in immediate changes to policies that favour industry.

 “*There’s little evidence to suggest that if you put these sorts of restrictions in place, that it will result in job losses. It’s a claim rather than anything that can be backed up by evidence. But that still had an impact”* (State government official).

####  Actors’ Ideas About Regulating Corporate Activity

 The neoliberal position of the Federal government that emphasizes free markets, deregulation, and individual choice sustained the self-regulation approach and undermined the scope to implement legislative restrictions on unhealthy food marketing. This sentiment was shared by technical experts, state/territory government officials and civil society actors.

 “*…they (i.e., the Federal government) have… neoliberal view of the sovereign consumer…and don’t understand the public health angle on all of this” *(Civil society actor).

 One of the key elements of the cultural-cognitive pillar is shared understanding and common knowledge of the issue by the actors nested within institutions. Two technical expert interviewees observed that industry actors and the Federal government share the same position regarding the regulation of corporate political activity, which contributes to industry self-regulation.

 “*…Liberal Party don’t even need to be lobbied…They totally think that it’s up to individuals to make a choice about whether they keep the TV on or they don’t” *(Technical expert).

 This was corroborated by a technical expert who formerly served as the top management at the State government. A legislative approach to policies was deemed as heavy-handed and highlighted the importance of public-private partnerships as an alternative.

 “*…legislative tools like banning something – or taxing something are highly effective*; *I do think there is an opportunity to work with other government departments and the private sector” *(Technical expert).

####  Perceptions and Understanding of Food Marketing as a Problem 

 Interviewees reported challenges with creating shared meaning and understanding of the harmful impacts of unhealthy food marketing on children. Health actors, who are often the lead advocates for policy reforms, perceived the scientific evidence as being difficult to present to other non-health policy-makers. The cited comment was the link between obesity and unhealthy food marketing was not seen as tangible. Additionally, singling the impact of food marketing intervention remained a challenge.

 “*…the difficulty always comes about the impact…for health advocates… we’re dealing with a much longer-term game. So, if the issue at hand is obesity, then restricting junk food advertising for starters is only one part of it – all the policy interventions need to happen” *(State government official).

 Understandings of food marketing as a policy issue were further complicated by counterevidence presented by the industry. Interviewee/(s) indicated that such evidence often created doubt about the effect of food marketing on obesity and questioned the need to implement a government regulation to restrict unhealthy food marketing. In addition, industry evidence on the economic impacts of regulation and loss of jobs was used and perceived to be easier for policy-makers to understand.

####  Framing Food and Policy Solutions

 Framing also emerged as one of the cultural-cognitive factors that influence policy change, including with respect to the foods that should/should not be advertised and to the framing of policy solutions. The framing of the policy solution was perceived to play an important role in getting politicians to prioritize food marketing. Government actors, technical experts and civil society interviewees identified framing strategies that would resonate with politicians and advance food marketing restrictions. These include framing food marketing policies as a means to protect vulnerable children and to bring in children’s rights and privacy.

 “*…if the framing is very much around protecting children, and that seems to be the most successful framing…” *(State government official).

 “*…that’s why we might start to bring in digital rights, human rights, and child rights…if we only focus on health, then it could be somewhat of a hindrance” *(Technical expert).

 The framing of food marketing regulation as a means to protect children was also used by the food and advertising industry, but its policy comprehensiveness remained arguable. For instance, the highly contested self-regulation and ACMA codes were framed as means to protect children but did not necessarily address the issue of exposure to unhealthy food marketing or regulate persuasive techniques used by advertisers beyond what is defined as “child-targeted.”

 “*The problem with the regulatory system at the moment is that it focuses on marketing that directly targets children, and that doesn’t really do anything about the fact that children are widely exposed to unhealthy food marketing that they find appealing and persuasive” *(Technical expert).

## Discussion

 This study explored institutional processes that shape current approaches for regulating the marketing of unhealthy food to children using Scott’s three pillars of institutions. Our interviews demonstrated that all three pillars – regulatory, normative, and cultural-cognitive influence the governance of food marketing by either sustaining the current practices that are dominated by self-regulation or creating pathways for policy change (statutory regulation of food marketing). Current approaches inhibit the development of strong statutory regulations by giving the impression that there is an existing system that protects children from food marketing despite the evidence showing that the current system is not adequate.^18,37^ Implementing self-regulation initiatives to derail or pre-empt government is a common strategy by commercial actors in the alcohol and tobacco industries.^38-40^ Past observations on the inhibitors of regulation of food marketing also found industry power to be one of the inhibitors of statutory regulations for food marketing in Australia^25^ and other countries such as Thailand,^41^ Fiji,^42^ and Canada.^43^ Furthermore, the governing of unhealthy food marketing regulation outside the jurisdiction of health inhibits the institution of statutory policies that specifically reduce unhealthy food marketing, particularly the delegation of marketing regulation to the communications department and the absence of food marketing in the food regulation system.

 Past failure attempts over the past decade to push for statutory food marketing regulations by civil society and public health advocates suggested the reluctance of the federal government to regulate unhealthy food marketing.^44^ This reluctance was also evident in the regulation of alcohol advertising and other nutrition policies, such as the Health Star Rating and food reformulation that are also implemented using non-legislative approaches in Australia.^45,46^ In the absence of statutory regulation specific to unhealthy food marketing, meta-regulation that involves third-party actors such as civil society overseeing aspects of regulation such as monitoring to increase the accountability of self-regulation initiatives is recommended.^47-49^ In the past, the federal government has been more open to third-party monitoring compared to outright statutory regulation.^44^ Studies show that even in the absence of statutory regulation, policies with robust standards and independent monitoring are effective.^50,51^ Therefore, strengthening the role of existing institutions and advocating for third parties to monitor current schemes is a potential pathway for improving the regulation of food marketing. However, this would require more participation of health actors and civil society as well as a more active oversight role by food agencies such as FSANZ and media regulatory agencies (ACMA).

 Interviewees from state/territory government departments of health highlighted elements of the normative pillar, such as norms for consultation and collaboration and institutional goals and priorities, as the ways to achieve statutory regulation of food marketing. Norms that prioritize a whole-of-government approach to nutrition/health (and therefore food marketing policy) as one of the policy levers were instrumental in getting food marketing regulations on the agenda for state and territory governments. However, this is not present at the federal government level, especially concerning the implementation of a strong statutory regulation in food marketing. While this is currently lacking, the interviewees noted a shift towards chronic diseases in the priorities of the regulation system. The shift in norms is an opportunity for food marketing regulation to get on the policy agenda. In the United Kingdom, the development of a statutory co-regulation approach by the Office of Communication was enabled by public health actors such as the Food Standards Agency and the Department of Health.^52^

 Framing, an element of the cultural cognitive pillar, is also influential in the food marketing policy space. Currently, there is a lack of a shared understanding of what constitutes food marketing. For example, marketing on food packages is a form of marketing according to the WHO classification of the forms of marketing^53^ and would ideally fall under the jurisdiction of FSANZ. However, FSANZ perceives its role in regulating food marketing as minimal despite having jurisdiction over on-pack labels. In addition, prioritizing food marketing among non-health actors who have jurisdiction to regulate, such as transport and communications, is a challenge because it often competes with economic interests that take priority under current institutional norms. Evidence from Chile shows that challenges of competing interests between departments can be solved by implementing one multi-pronged food act that addresses nutrition labelling, marketing in schools and marketing on broadcast and non-broadcast media.^16,52,54^ *WHO Regional Action Framework on Protecting Children from the Harmful Impact of Food Marketing* also underscores the importance of multiple policy instruments, multi-sectoral collaboration, and the engagement of various stakeholders to achieve sustainable and effective solutions in protecting children from the harmful effects of food marketing.^55^

 While using the vulnerability of, and the need to protect, children is a common understanding across different actors, the focus of industry codes and the ACMA on “child-directed” marketing^56^ is not the same as the WHO recommended “reduction of exposure to unhealthy food marketing.”^53^ This suggests that different actors strategically use a vulnerability framing that aligns with their interests, which conflict with public health recommendations. There is, therefore, a disconnect between what industry or government actors want to do, which is to protect children (cultural cognitive pillar) and how it ought to be done (normative pillar). The exploitation of public health framing is a frequently used strategy to influence policies by unhealthy commodity industries such as alcohol and tobacco.^57,58^

###  Limitations 

 While this study provides a theory-informed novel analysis of institutional factors that constrain and advance policy change, it was subject to some limitations. First, the analysis of how actors exercise agency within the institutions they work for was not examined, as the interviewees were asked questions as representatives of their institutions. Secondly, due to the nature of unhealthy food marketing as a policy issue, more interest was observed from health-oriented actors and poor responses from actors such as transport, education, and communication, who also have a role in regulating unhealthy food marketing. Examining how the three pillars transpire in non-health actors would advance the understanding of institutional processes that shape food marketing regulation.

## Conclusion

 This study examined and identified institutional factors that constrain policy change and potential pathways to comprehensive statutory regulation of food marketing. The findings highlight a need for the rearrangement of institutions that regulate unhealthy food marketing to increase the role of the Department of Health to ensure that the regulation of unhealthy food marketing centres health. Intersectoral collaboration and institutional goals that take a whole-of-government approach, which have been identified as enablers at the state/territory government level, should be amplified, and ways of adopting similar approaches at the Federal government level should be explored. Lastly, the findings may be useful for helping civil society actors design advocacy strategies that are targeted at specific institutional factors that inhibit the comprehensive regulation of marketing.

## Ethical issues

 Ethics approval was obtained from the Australian National University Human Research Ethics Committee (2019/222).

## Competing interests

 Authors declare that they have no competing interests.

## Funding

 The project was funded by the Australian Research Council Discovery project (ARCDP 190100576).
